# Factors Affecting Trypanosome Maturation in Tsetse Flies

**DOI:** 10.1371/journal.pone.0000239

**Published:** 2007-02-21

**Authors:** Ewan Thomas Macleod, Alistair Charles Darby, Ian Maudlin, Sue Christina Welburn

**Affiliations:** Centre for Infectious Diseases, College of Medicine and Veterinary Medicine, The University of Edinburgh, Easter Bush Veterinary Centre, Roslin, Midlothian, United Kindgom; Federal University of São Paulo, Brazil

## Abstract

*Trypanosoma brucei brucei* infections which establish successfully in the tsetse fly midgut may subsequently mature into mammalian infective trypanosomes in the salivary glands. This maturation is not automatic and the control of these events is complex. Utilising direct *in vivo* feeding experiments, we report maturation of *T. b. brucei* infections in tsetse is regulated by antioxidants as well as environmental stimuli. Dissection of the maturation process provides opportunities to develop transmission blocking vaccines for trypanosomiasis. The present work suggests L-cysteine and/or nitric oxide are necessary for the differentiation of trypanosome midgut infections in tsetse.

## Introduction

Trypanosomes of different species undergo cycles of development of varying complexity within the tsetse fly, transforming from bloodstream forms to procyclic non-mammalian infective forms in the fly midgut. To complete their life-cycle *Trypanosoma brucei brucei* and the causative organisms of human African trypanosomiasis, *T. b. rhodesiense* and *T. b. gambiense*, must migrate from the midgut of the fly and transform to infective metacyclic forms in the salivary glands. Only a proportion of midgut infections mature into salivary gland infections and the process is sex limited (not controlled by a sex-linked gene), with male tsetse maturing significantly more midgut infections than females [1], [2]. Trypanosome genotype also influences maturation; trypanosomes resistant to human serum (*T. b. rhodesiense*) are less likely to produce mature infections than human serum sensitive (*T. b. brucei*) parasites [3]. Previous reports showed that interactions with tsetse lectins were important in the development process. Blocking of these lectins with specific sugars increased midgut infection rates [4], however, continuous feeding of glucosamine significantly lowered the maturation rates of established midgut infections [5]. Removal of serum from the bloodmeal has a similar effect, increasing midgut infection rates but reducing the number of established midgut infections maturing [6]. Apart from effects of fly sex, sugars and trypanosome genotype there is little indication that maturation may be manipulated experimentally. However, it has been shown that the number of trypanosomes per fly gut remains remarkably constant once infection is established [7], [8] suggesting some form of regulation of parasite numbers. It has also been shown that the number of trypanosomes imbibed with the infective bloodmeal does not influence maturation of midgut infections [9] and that one trypanosome is sufficient to infect the midgut of a susceptible fly [10].

We have recently shown that antioxidants can inhibit death of *T. b. brucei* in the midgut of tsetse flies suggesting that reactive oxygen species (ROS) play a major role in killing incoming trypanosomes [11]. Here we describe the effects of antioxidants and environmental change on the maturation of *T. b. brucei* infections in tsetse flies.

## Results

To enhance midgut infections to a level at which changes in rates of maturation would be apparent, infective feeds were supplemented with 100 µM 8-Br-cGMP [12] unless otherwise stated.

**Figure 1:**
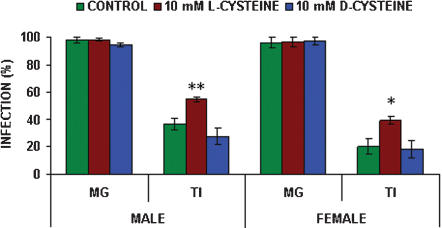
Effect of cysteine on transmission of *T. b. brucei* in male and female *G. m. morsitans*. All flies were infected at their first feed, the infective bloodmeal containing 100 µM 8-Br-cGMP. Flies were maintained on blood containing either 10 mM L- or D-cysteine from the second feed. Control flies were not fed cysteine. Data presented as the mean +/− S.E.M from three experiments. (MG midgut infections; TI proportion of midgut infections maturing to salivary gland infections). Significance: ***p<0.001; ** *p*<0.01; *p<0.05 versus the corresponding control values.

The effects of the subsequent addition and thereafter continuous feeding of 10 mM L- or D-cysteine on maturation of *T. b. brucei* in *G. m. morsitans* are shown in Figure 1. No significant differences in midgut infection rates were found in either male or female *G. m. morsitans* with the subsequent addition of either 10 mM L- or D-cysteine when compared with controls which received no cysteine (compared to control: male: *p* = 0.162; female: *p* = 0.801). Midgut infection rates for males fed L- or D-cysteine were 98% (n = 113) and 94% (n = 85) respectively compared to the control value of 98% (n = 106). Midgut infection rates for females fed L- or D-cysteine were 96% (n = 85) and 97% (n = 68) respectively compared to the control value of 98% (n = 106). However, L-cysteine significantly increased the proportions of midgut infections maturing into salivary gland infections in both male (*p* = 0.009) and female (*p* = 0.011) tsetse (expressed as transmission index, TI% [3], calculated as the proportion of salivary gland infections maturing from established midgut infections). L-cysteine increased salivary gland infections in males from 37% (n = 104) to 55% (n = 111) and in females from 20% (n = 69) to 39% (n = 82). Addition of D-cysteine resulted in TI of 28% (n = 80) in males and 18% (n = 66) in females, not significantly different from the control (male: *p* = 0.155; female: *p* = 0.811). Differences in TI between the additions of L- or D-cysteine were significant in both males (*p*<0.001) and females (*p* = 0.007).

**Figure 2:**
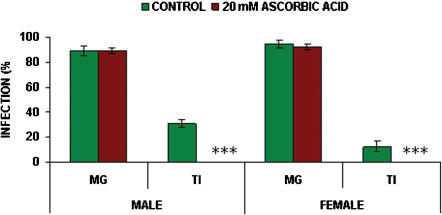
Effect of ascorbic acid on transmission of *T.b. brucei* in male and female *G. m. morsitans*. All flies were infected at their first feed with, the infective bloodmeal containing 100 µM 8-Br-cGMP. Flies were maintained on blood containing 20 mM ascorbic acid from the second feed. Control flies were not fed ascorbic acid. Data presented as the mean +/− S.E.M from three experiments. (MG midgut infections; TI proportion of midgut infections maturing to salivary gland infections). Significance, *p* values as in Figure 1.

The effects of the subsequent addition and thereafter continuous feeding of 20 mM ascorbic acid on transmission of *T. b. brucei* in *G. m. morsitans* are shown in Figure 2. No significant differences in midgut infection rates were found in either male or female *G. m. morsitans* with the subsequent addition of 20 mM ascorbic acid when compared with controls which received no ascorbic acid (male: *p* = 0.817; female: *p* = 0.515). Midgut infection rates for male flies were both 89% (control, n = 92; ascorbic acid, n = 83). Midgut infection rates for female flies were 95% (n = 92) and 92% (n = 92) for controls and those fed 20 mM ascorbic acid respectively. Ascorbic acid significantly decreased the proportions of midgut infections maturing into salivary gland infections from a control value of 30% (n = 82) to 0% (n = 74) in males (*p*<0.001) and from 13% (n = 87) to 0% (n = 85) in females (*p*<0.001).

**Figure 3:**
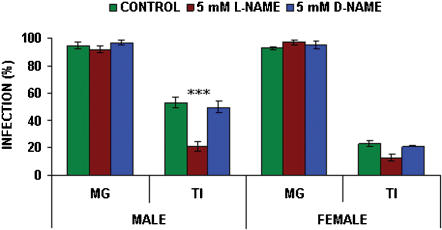
Effect of NAME on transmission of *T.b. brucei* in male and female *G. m. morsitans*. All flies were infected at their first feed, the infective bloodmeal containing 100 µM 8-Br-cGMP. Flies were subsequently maintained on blood containing either 5 mM L- or D-NAME. Control flies were not fed NAME. Data presented as the mean +/− S.E.M from three experiments. (MG midgut infections; TI proportion of midgut infections maturing to salivary gland infections). Significance, *p* values as in Figure 1.

The effects of the subsequent addition and thereafter continuous feeding of 5 mM L- or D- N-nitro-arginine-methyl-ester (NAME) on transmission of *T. b. brucei* in *G. m. morsitans* are shown in Figure 3. No significant differences in midgut infection rates were found in either male (*p* = 0.338) or female (*p* = 0.438) *G. m. morsitans* with the subsequent addition of either 5 mM L- or D-NAME when compared with controls which received no NAME. Midgut infection rates for males fed L- or D-NAME were 92% (n = 98) and 97% (n = 95) respectively compared to the control value of 95% (n = 91). Midgut infection rates for females fed L- or D-NAME were 97% (n = 96) and 95% (n = 99) respectively compared to the control value of 93% (n = 99). L-NAME significantly reduced (*p*<0.001) the proportions of midgut infections maturing into salivary gland infections from a control value of 53% (n = 86) to 21% (n = 90) in males. There was no significant effect of D-NAME when compared to controls which showed TI values of 50% (n = 92; p = 0.623). Differences in TI between the additions of L- or D-NAME were significant (*p*<0.001) in male flies. There was no significant effect of NAME on maturation rates in female flies (*p* = 0.162) with control flies showing TI values of TI of 23% (n = 96) compared to 13% (n = 93) and 21% for flies fed L-NAME or D-NAME respectively.

**Figure 4:**
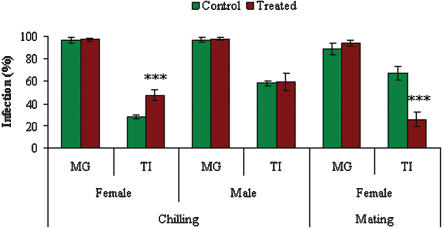
Effect of chilling and mating on transmission of *T. b. brucei* in *G. m. morsitans*. All flies were infected with *T. b. brucei*, the infective bloodmeal containing 15 mM GSH. For investigation of chilling treated flies were chilled (4°C for 30 min) three days post infection, control flies were not chilled. For investigation of mating, treated flies were mated immediately post infective feed for three days. Both groups in the mating experiment were chilled to allow separation of male flies, control flies were not mated. Data presented as the mean +/− S.E.M from four experiments. (MG midgut infections; TI proportion of midgut infections maturing to salivary gland infections). *p* values as in Figure 1.

The effects of temperature on female and male *G. m. morsitans* and of mating on female *G. m. morsitans* transmission of *T. b. brucei* are shown in Figure 4. To enhance midgut infections to a level at which changes in rates of maturation would be apparent, the infective feed was supplemented with 15 mM glutathione (GSH). Neither chilling (male: *p* = 0.678; female: *p* = 0.579) nor mating (*p* = 0.231) had any significant effect on midgut infections. Midgut infections in male flies were 97% (n = 99) in control flies (not chilled) compared to 98% (n = 97) in flies that had been chilled. Midgut infections in female flies were 96% (n = 131) in control flies (not chilled) compared to 97% (n = 147) in flies that had been chilled. Midgut infections in female flies were 89% (n = 99) in control (not mated) flies compared to 94% (n = 97) in flies that had been mated. Female flies chilled (30 min at 4°C) three days post infection matured significantly more (*p*<0.001) midgut to salivary gland infections than flies that were not chilled. TI values for control flies were 28% (n = 126) compared to 48% (n = 143) for chilled flies. Chilling female flies for up to three hours did not result in further increases in maturation (data not shown). Chilling of male flies had no significant effect (*p* = 0.770) on maturation rates, TI values for control flies were 58% (n = 91) compared with 60% (n = 82) for chilled flies. Females that were mated produced significantly fewer (*p*<0.001) salivary gland infections than unmated females. TI values for control flies (unmated) were 67% (n = 88) compared with 25% (n = 91) for mated flies.

The following compounds had no significant effect on maturation when flies were infected with 100 µM 8-Br-cGMP: 15 mM GSH, 10 mM N-acetylcysteine (NAC), 20 mM proline, 5 mM cystine, 10 mM uric acid, 15 mM L-arginine and 15 mM D-arginine (data not shown). Continuous feeding of 15 mM GSH until dissection had no effect on rates of maturation.

## Discussion

The process by which trypanosomes move from the midgut to the salivary glands of tsetse flies has been shrouded in mystery for over one hundred years. There has been little experimental work on the differentiation of *T. brucei* from non-mammalian infective procyclic midgut forms to mammalian infective metacyclic forms in tsetse as this maturation process naturally occurs at very low levels. Removal of serum from the tsetse diet was shown to inhibit maturation [6] and feeding glucosamine to tsetse for as little as five days also lowered maturation rates, suggesting that the signal to mature is received within the first few days of the trypanosomes entering the fly midgut [5], [13].

In the current work addition of cysteine to the fly diet did, however, provide an insight into maturation processes (see Figure 1). L-cysteine significantly increased maturation rates in both male and female tsetse while the non-physiological isomer D-cysteine had no effect when compared with control flies, suggesting that L-cysteine may be utilised by an enzyme as a substrate [14], [15]. Bloodstream form trypanosomes cannot utilise cystine but provision of cystine in co-culture of trypanosomes with insect cells results in the release of cysteine by the insect cells [15]. In the present work cystine did not affect maturation suggesting that L-cysteine is being used directly by trypanosomes to promote maturation. The failure of NAC to influence maturation rates suggests that trypanosomes do not possess the ability to de-acetylate NAC. Continuous feeding of GSH had no effect on maturation rates, suggesting that it is not broken down into its component parts in the fly, thereby releasing L-cysteine.

Ascorbic acid was the only compound tested that acted in a manner similar to glucosamine [5], i.e. increasing susceptibility to midgut infection while reducing maturation rates; continuous addition of ascorbic acid to the bloodmeal from the second feed completely blocked maturation (see Figure 2). Recently it has been shown that glucosamine can scavenge superoxide and hydroxyl radicals [16]; this offers an alternative explanation for observed increases in midgut infection rates and reductions in rates of maturation previously thought to be linked to inhibition of trypanocidal lectins by glucosamine [4]. Since L-cysteine increased maturation rates and other antioxidants, including D-cysteine and GSH, had no such effect, it would suggest that the inhibition of maturation observed with ascorbic acid and glucosamine [4] is independent of the oxidative state of the midgut environment or possibly a specific oxidant is involved in triggering maturation. Previous work [17] had shown that L-NAME, a nitric oxide synthase inhibitor, did not increase tsetse susceptibility to midgut infection with trypanosomes; however, in the present work continuous feeding of L-NAME significantly reduced maturation rates of established midgut populations in male tsetse (see Figure 3). The experiments presented here with L-NAME suggest that NO may be involved in the maturation process; supplementation of the diet with L-arginine, the precursor of NO, did not affect maturation rates in the present work; if trypanosomes are responding to NO, then the concentration would be crucial.

Environmental stresses, both external and internal may affect the differentiation of trypanosomes in the tsetse fly. In the wild, temperatures can drop significantly during the night in southern Africa [18] and sleeping sickness epidemics have been linked to seasonal temperature periodicity [19]. In the laboratory the most important factor in differentiation of trypanosomes from bloodstream to procyclic forms is a temperature drop from 37°C to 26°C [20]. Previous work had shown that chilling of tsetse at 0–5°C for 30 minutes post infection increased midgut infections but did not significantly increase proportions of trypanosome infections maturing in male tsetse [9]; keeping tsetse at 20°C throughout their lives blocks maturation of *T. brucei* [21]. In the present work severe chilling for a short period had no effect on midgut infection rates (although they had been boosted by supplementation of the bloodmeal with GSH) but significantly increased maturation of trypanosome infections in female flies (see Figure 4). The fact that temperature shock did not increase rates of maturation in males suggests that there is a natural limit to maturation indices which are normally reached in male flies or the factor/s which inhibit maturation in females are negated by chilling. Chilling has been shown to induce the synthesis of heat shock proteins in Drosophila [22] and the effect of chilling in tsetse may follow similar patterns. The production of these heat shock proteins or the cold shock itself may have some effect on the trypanosomes, potentiating transmission.

In the wild, female tsetse will invariably be inseminated within a few days of emergence. Experiments investigating maturation rates in female tsetse normally involve unmated flies; the present work has shown, however, that a mated female is less than half as likely to mature infection a midgut infection as an unmated female (see Figure 4). Mated female tsetse fed on animals infected with *T. vivax, T. congolense* or *T. brucei* show no significant differences in fitness parameters such as number of pupae produced or pupal weight when compared to control flies fed on uninfected animals [23]. While a trypanosome infection does not affect the reproductive fitness of the fly, tsetse reproduction clearly has a detrimental effect on maturation of trypanosome infections. This could be due to a reduction in the amounts of free nutrients available to trypanosomes which will instead go towards larval production while levels of circulating hormones will also be different in pregnant tsetse. Although levels of cyclic nucleotides fluctuate during pregnancy in tsetse [24] feeding of 8-Br-cGMP or 8-Br-cAMP had no effect on maturation rates [12].

The pathway taken by trypanosomes through the tsetse fly is complicated, evidenced by the low infection rates found in flies even in endemic areas [25]. Similar bottlenecks face malaria parasites as they complete development in the mosquito some of which have been shown to involve increases in oxidative stress [26]. Oxidative stress has also been shown to be involved in immune defences in other insects including Drosophila [27] and *Rhodnius prolixus* [28]. We have recently shown that antioxidants greatly increase midgut infection rates of trypanosomes in tsetse [11] suggesting a role for oxidative stress in refractoriness of tsetse to trypanosome infection. However, taken together with the present work, it appears that either the oxidative state of the tsetse midgut plays no part in maturation or that a specific oxidant (which could for example be knocked out by ascorbic acid) may be involved in triggering migration to the salivary glands.

In conclusion, the work presented here suggests that, having established themselves as a replicating population in the tsetse midgut, trypanosomes may require a NO signal and/or the presence of L-cysteine to promote migration to the salivary glands and maturation into mammalian infective forms.

## Materials and Methods

### Fly infections

*G. m. morsitans* were infected on the day following the day of emergence from the puparium with bloodstream form *T. b. brucei* stock Buteba 135 which was isolated from a cow in Buteba village, Uganda in 1990. Infective feeds were given *in vitro* using thawed stabilates of trypanosomes suspended in defibrinated ovine blood with trypanosome concentrations between 1×10^6^ to 4×10^6^ given for all infective feeds [29]. Flies which did not feed were removed from the experiment. Following infection, flies were maintained at 25°C unless otherwise stated and 70% relative humidity and fed on defibrinated ovine blood through an artificial membrane. Flies were dissected 28 d post infection and midgut and salivary glands examined for the presence of trypanosomes by phase-contrast microscopy (×400).

### Feeding of test compounds

*G. m. morsitans* were infected with a bloodmeal supplemented with 100 µM 8-Br-cGMP and then were subsequently fed defibrinated ovine blood supplemented with one of the following compounds in saline: GSH, NAC, L- and D-cysteine, uric acid, ascorbic acid, glutamate, cystine, L- and D- N-nitro-arginine-methyl-ester (NAME), L- and D-arginine, proline (all supplied by Sigma UK). Control flies received 100 µM 8-Br-cGMP with the infective feed and were then fed blood supplemented with saline only.

### Temperature effects

*G. m. morsitans* were infected with a bloodmeal supplemented with 15 mM GSH; three days post-infection, flies were chilled for 30 min at 4°C then returned to 25°C. Control flies received no cold shock.

### Mating effects

Female *G. m. morsitans* were infected with a bloodmeal supplemented with 15 mM GSH. Immediately after feeding, one group of females were allowed to mate with males (10 d old) for three days. Females were then chilled for 30 min at 4°C and separated from their mates; unmated control flies were chilled in the same way as mated flies.

### Statistical analysis

To examine if test compounds had a significant effect on midgut infection rates, generalised linear models with binomial errors were used on the proportion of flies infected, with the effects of replicates taken into account by entering replicate into the model first. Analyses were carried out in a two-stage process: first, overall differences between controls and treatments were considered and, if these were significant, then each compound was compared with its control in post-hoc testing. All analyses were carried out in R version 1.9.1 ((c) R project) and *p*<0.05 was taken to indicate significance. To examine if compounds had a significant effect on maturation rates the same analysis was carried out as for midgut infection rates except that the number of mature infections from flies with midgut infections was used for analysis
